# Youth Perspectives of Neglect Signs and Help-Seeking

**DOI:** 10.3390/bs14080704

**Published:** 2024-08-12

**Authors:** Ayala Cohen, Ibtisam Marey-Sarwan, Daphna Gross Manos

**Affiliations:** 1 Social Work Department, Tel Hai College, Qiryat Shemona 1220800, Israel; grossdaphna@telhai.ac.il; 2 Early Childhood Department, Sakhnin Academic College, Sakhnin 3081000, Israel; ibtisam.marey@sakhnin.ac.il

**Keywords:** child neglect, youth perspective, neglect signs, assistance factors

## Abstract

Child neglect, recognized as the most prevalent form of child maltreatment with profound repercussions on children’s development, has received limited scholarly attention compared to abuse. The current study addresses this shortfall with a qualitative research investigation involving 10 multicultural focus groups of youths aged 12 to 15. The research examined how young individuals identify signs of child neglect and discern whether formal and informal sources of assistance may be relied upon to assist in addressing this issue. Through qualitative–thematic analysis, three primary themes emerged: (1) Characteristics of neglected children, (2) challenges in identifying child neglect, and (3) official and unofficial sources to appeal for assistance when child neglect is identified. This study’s insights concern peers’ recognition of signs indicating neglect in children and their perspectives on potential assistance.

## 1. Introduction

Child neglect is a serious global problem [1] (Kobulsky et al., 2020) with negative physical, psychological, and social consequences [2] (Merrild & Frost, 2021). The well-being, development, social skills, and achievements, as well as their physical and mental health may be impacted throughout life [3] (Turner et al., 2019). The features of child neglect vary by age. Infants and young children, reliant on caregivers, are highly susceptible to neglect, while more independent adolescents show different signs and risks. Understanding these differences is key for effective identification and intervention. This study focuses on adolescents’ perceptions of neglect, offering insights into their identification and the factors they consider relevant in addressing it.

In 2021, it was estimated by the U.S. Department of Health & Human Services [4] that 86% of all maltreatment-related deaths were due to neglect. In 2021, there were 50,010 children under a child protection plan in England and Wales, with neglect constituting 52% of the initial child protection plans [5] (Department for Education, 2021). In Israel, where the current study took place, the Israel National Council for the Child [INCC] [6] cited neglect as the main reason for a child’s at-risk status in 31% of cases reported in Israel in 2018. 

Child neglect poses a challenge to both the care systems and the public [7] (Daniel, 2015). The extensive nature of child neglect, its severity, and enduring repercussions necessitate a proactive approach. It is imperative to enhance investment in the development of strategies for detection, measurement, prevention, and intervention [2,8] (Han et al., 2024; Merrild & Frost, 2021). Several issues seem to have limited such prevention efforts to date. One is the fact that professional practice and academia have introduced a wide range of child neglect definitions; the absence of a clear definition impedes the precise and accurate measurement of this phenomenon [9,10] (Lui et al., 2022; Haworth et al., 2024). Another complexity is that the identification of neglect is heavily reliant on the subjective perspective of professional workers, leading to considerable variability [11] (Elias et al., 2018). In fact, professionals have stated that they often do not report child neglect, especially emotional neglect, because many cases do not seem “serious enough” to report [12] (Bullock et al., 2019). Although progress has been made in establishing essential child protection and other safeguards against neglect in most countries, significant barriers and inadequacies remain. Much work remains to better assess and address this serious problem in every country [1] (Kobulsky et al., 2020).

Neglect could be prevented or halted through community engagement in identification and early support [13] (Arimoto & Tadaka, 2021). However, according to a recent literature review [14] (Lafantaisie et al., 2020), conventional studies on neglect typically disregard the genuine experiences and viewpoints of parents and children. Therefore, the current study seeks to reveal this crucial knowledge by asking youths about the indicators they use to identify child neglect and to understand whom they approach for assistance in such situations.

## 2. Literature Review

### 2.1. Definitions of Child Neglect

Neglect, regarded as a subtype of child maltreatment [11] (Elias et al., 2018), may be physical, medical, educational, emotional (including a lack of affection and empathy), environmental, and supervisory [15,16] (Julien et al., 2019; Morrongiello & Cox, 2020). The United Nations Convention on the Rights of the Child (CRC) acknowledges various forms of neglect—physical neglect, emotional neglect, neglect of physical and mental health, educational neglect, and abandonment [1] (Kobulsky, et al., 2020).

The Israel Ministry of Welfare and Social Services [17] defines child neglect as, among other factors, insufficient hygiene, inappropriate clothing for the season or improper sizing, inadequate supervision, delays in seeking medical treatment leading to repeated hospital visits, lag in physical development manifested through poor weight and nutrition, and the child’s involvement in prohibited and dangerous substances like alcohol and drugs. The *Mandatory Reporting Law* in Israel requires anyone to report suspected child maltreatment by a parent or caregiver to the police or a child protection social worker. Professionals working with children face up to six months in prison for non-compliance (Penal Code, Amendment no. 26, 1989). The Israeli Department of Education’s 2008 guidelines mandate that teachers consult with school counselors to establish reporting protocols, decide on informing parents, and report the abuse.

A recent study of health professionals, educators, welfare workers, and police officers reported that these service providers found it challenging to define child neglect [2,18,19] (Alyoubi et al., 2024; Grégoire-Labrecque et al., 2020; Merrild & Frost, 2021). Adopting a child-centered definition of neglect could embrace a wide range of potentially detrimental circumstances influenced by various levels of social ecology, including factors beyond parental control. This inclusive approach aims to account for diverse situations globally [20] (Proctor & Dubowitz, 2014).

### 2.2. Child Neglect Prevalence

Neglect is difficult to define conceptually and operationally and, therefore, to measure [21] (Dubowitz et al., 2005). A meta-analysis estimated the worldwide prevalence of neglect to be 16% for physical neglect and 18% for emotional neglect [22] (Stoltenborgh et al., 2013). In the United States, neglect makes up 75% of maltreatment reports to Child Protective Services (CPS). It is four times more common than physical abuse and nearly nine times more common than sexual abuse [23] (United States Department of Health and Human Services [USDHHS], 2019). According to UNICEF data, approximately one-sixth of preschool children worldwide encounter supervisory neglect and one-third lack early-learning support [1] (Kobulsky et al., 2020).

### 2.3. Child Neglect Identification

“Neglect” can be such a wide-ranging conceptual and intangible matter that it defies clear identification and definition. This situation makes it challenging to build a clear guide for professionals to identify the phenomenon and respond to its victims [24] (Hamarman et al., 2002).

One concern about the complexity of child neglect is that it is a continuous variable [25] (DeLong-Hamilton et al., 2016) where the quality of care can vary from excellent to markedly deficient; that is, a continuum exists spanning from the complete satisfaction of a child’s basic needs to his/her complete neglect [21] (Dubowitz et al., 2005). Another identification challenge is the dependence on professional workers’ subjective perspectives, which tend to be highly variable [11] (Elias et al., 2018). As Horwath [26] (2007) notes, “practitioners’ feelings, experiences, values and beliefs routinely influence practice” (p. 1299).

Still, common indicators of child neglect may include inadequate clothing, poor hygiene, hunger, persistent tardiness, absence, or cognitive or emotional developmental delays [27] (Daniel, 2008). Child neglect can also show more socio-emotional signs reflected by the child’s behavior. In a systematic review, Maguire et al. [28] (2015) found child neglect to be associated with externalizing and internalizing behaviors, an inability to regulate emotions, lower academic skills, lower self-esteem, depression, and difficulty initiating or maintaining friendships.

Emotional neglect is challenging for professionals to detect, and the focus and concerns have been on poor living conditions, hygiene, nutrition, and clothing [29] (Brandon et al., 2014). Research indicates emotional neglect is linked to child psychiatric disorders [30] (Young et al., 2011) and depression [31] (Webb et al., 2007). Additionally, social workers often view psychological neglect as more harmful than physical neglect [32] (Bernard, 2019).

Research shows that a child’s age significantly impacts the risk of neglect. Infants and young children, who depend entirely on adults for their basic needs, are highly vulnerable to neglect, leading to developmental delays [33] (Akehurst, 2015), health issues [34] (Bullinger et al., 2020), and cognitive, linguistic, and behavioral difficulties [35] (Spratt et al., 2012). 

Compared to infants and young children, adolescents face different neglect challenges due to their increased independence. They can suffer from emotional distress, academic problems, domestic violence, mental health issues, learning disabilities, substance abuse, and other risky behaviors [36] (Stevens & Laing, 2015). Neglect in adolescents is linked to antisocial behavior, delinquency, exposure to deviant peers, and violent behavior [37] (Braga et al., 2017). To effectively address neglect requires recognizing these age-specific differences and risks. 

Since child neglect can have profound psychological and emotional consequences for children and youth, the need to understand their subjective experiences intensifies [38] (Hildyard & Wolfe, 2002). 

### 2.4. Reporting and Assistance Services

The UN’s CRC mandates that all countries who are signatories must establish comprehensive child protection systems to guarantee a unified approach to addressing instances of child abuse and neglect [39] (Svevo-Cianci et al., 2010). 

Numerous countries have laws and policies addressing neglect, but their implementation remains uncertain, posing a common challenge even in high-income nations. Similarly, many countries report the existence of services aimed at addressing and preventing child maltreatment that are sadly lacking in necessary capacity and quality [1] (Kobulsky et al., 2020). 

In the United States, people can report neglect if they suspect it. However, the definition of “mandatory reporters” can vary in 50 states. Typically, physicians, social workers, educators, mental health professionals, childcare providers, medical examiners, and police officers are considered to be mandatory reporters [40] (DePanfilis, 2006a). 

Israel’s *Mandatory Reporting Law* mandates that all citizens must promptly report any suspicion of child maltreatment by a parent or guardian to the police or a designated social worker with legal authority. Failure to comply with this obligation can result in professionals who work with children and youth facing severe penalties, including imprisonment for up to six months [41] (Penal Code, Amendment no. 26, 1989). 

The absence of agreed-upon and clear definitions leads to differences between countries in the laws that they enact and, subsequently, to differences in the scope of reporting on children who have experienced abuse and neglect [24] (Hamarman et al., 2002). Levi et al. [42] (2015) found significant variability in how childcare providers interpret “reasonable suspicion”, raising the question of whether there is any consistency in the threshold for applying mandated reporting.

Professionals have a crucial role in supporting children and families with a unique opportunity to intervene for a child’s well-being and safety. They do, however, face various challenges when dealing with suspected cases of child abuse and neglect (CAN) [43,44] (Pietrantonio et al., 2013; Wilson & Lee, 2021), such as insufficient standardized training in identifying and managing CAN incidents and a lack of clarity on the procedures for reporting to CPS as required by law. Additionally, professionals may hesitate to address maltreatment concerns directly with the child’s parents or caregivers due to fears of potential consequences for the child or the professionals themselves [43] (Pietrantonio et al., 2013). In their literature review, Wilson and Lee [44] also refer to obstacles regarding resources and support, sociocultural context, reporter traits, and psychological attributes.

Frequently, children serve as the primary source of information in cases of child abuse and neglect, yet various motivational and developmental barriers can impede effective interviews with them. The few studies that have reported on interviews conducted with children have proven highly valuable [45,46,47,48] (Gross-Manos et al., 2023; Marey-Sarwan, 2019; Marey-Sarwan et al., 2023; Testa & Poertner, 2010). In their study of children’s testimonies of parental neglect, Lavi and Katz [49] (2016) revealed distinct patterns that illuminated the challenges children face in such circumstances. Children who struggle to disclose instances of neglect are often influenced by their fear and sadness about potential interventions by external figures. These findings underscore the urgency of dedicating additional resources to support families and communities in addressing and preventing child maltreatment, with particular emphasis on addressing child neglect.

The literature on child neglect illustrates the challenges for professionals in identifying this phenomenon when encountering children and families at risk and the subsequent complexity of reporting to child protection authorities. This situation exposes children’s safety to risk and requires support from others o interact with them, including their peers. 

### 2.5. Youths’ Perspective on Child Neglect

The perspectives of children and youth on neglect are critical when focusing on children’s needs and may contribute significantly to the definition of child neglect [50,51] (Christ et al., 2017; Straus & Kantor, 2005). Professionals’ failure to sufficiently or effectively engage with children or young people on the issue has led to the phenomenon commonly known as the “invisible child” [52] (Ferguson, 2017). This failure emphasizes the need to better understand different perspectives regarding child neglect and specifically take a child-focused approach to understand the perceptions of children and youth [50] (Christ et al., 2017). A recent literature review identified that mainstream studies on neglect generally exclude the lived experiences and points of view of parents and children and thus: “appear to be insufficient for an optimal understanding of the situation of families” [14] (Lafantaisie et al., 2020, p. 425).

Munro [53] (2011) emphasized the unique insights that young people can provide as they are witnesses to the impacts of neglect. Failing to consider their viewpoints risks overlooking crucial details or misinterpreting the gravity of neglectful behaviors [54] (Cossar et al., 2011). 

Generally, theoretical discourse indicates that children are knowledgeable regarding their worldviews and the contexts in which they live, thus their views must be heard [45,55,56] (Clarke et al., 2011; Mayall, 2015; Marey-Sarwan, 2019). Only limited research has specifically addressed perceptions of child neglect by children and youth [57] (Gorin, 2016). Choi and Thomas [58] (2015) conducted a study asking Korean children and parents to assess the severity of statements related to various domains of child neglect. The findings revealed that children tended to perceive child neglect situations as more serious than their parents, with notable differences across several domains.

In qualitative research conducted in the UK, Gorin [57] (2016) held focus groups with 51 young people known to the welfare system, all of whom had experienced child neglect. The study highlighted distinct perceptions and definitions of neglect among young people as compared to adults. Most participants viewed neglect as a manifestation of inadequate parental responsibility and care. Finally, Lavi and Katz [49] (2016) examined forensic investigations involving young children in Israel, focusing on cases indicative of a high likelihood of neglect. The study explored how children narrated their experiences and understood neglect. The findings underscored the challenges that the children faced in verbalizing neglect and the internal conflict they experienced during investigations, prompting many to attempt to explain their parents’ behavior. 

The current study’s authors conducted previous studies) this article is part of the same project) about the perceptions of neglect among youths from various population sectors in our country (according to gender, degree of religiosity, ethnicity, socio-economic status, type of settlement, etc.). They found similarities between the definitions and descriptions of neglect by children across different population groups. However, the participants added a new dimension to the conceptualization known in the literature—the emotional context that is lacking in neglectful behaviors [45,47] (Gross-Manos et al., 2023; Marey-Sarwan et al., 2023). Therefore, children may be more attuned to subtle forms of neglect, such as emotional neglect or inadequate supervision, which can be challenging for adults to recognize [7] (Daniel et al., 2015).

## 3. Methods

In this research, a qualitative paradigm was utilized to encompass complicated and diverse perspectives on the phenomenon of child neglect. The objective was to attain a comprehensive understanding by exploring the participants’ subjective interpretations and examining their perceptions and worldviews [59] (Denzin & Lincoln, 2011).

### 3.1. Participants

Information was collected through 10 focus groups involving 63 Jewish and Arab Israeli youth aged 12 to 15, including 34 girls and 29 boys from the northern region of Israel. Six of the groups involved Jewish youth from both urban and non-urban settings. Two of the six groups included more religious youth, and one focus group occurred in a center for at-risk youth. The remaining five groups involved Arab youth, including Muslims and Druze participants.

No specific social demographic data, such as parental income or education, were collected due to the qualitative nature of the research and the priority given to maintaining participant confidentiality and anonymity. However, the study did aim to include a diverse range of participants based on the socioeconomic index of their places of residence and types of settlements to represent a variety of normative family backgrounds. This approach was intended to provide a broad perspective on the issues of neglect.

It is important to note that we did not have prior information on whether the group participants had experienced neglect.

### 3.2. Data Collection

Utilizing focus group discussions is a standard method to foster dialogue and debate on a research topic that necessitates collective perspectives and the opportunity to explore the meanings inherent in those views and incorporate participants’ experiences and beliefs [60] (Nyumba et al., 2018). Each focus group discussion included four to six participants and lasted approximately one hour. The sessions were led by one of the authors and a research assistant in the participants’ native language (Hebrew or Arabic). The participants were recruited through community education after-school programs for youth. These programs issued a call for participants, and for those who consented, focus group sessions were arranged at the program’s location or in their residence. It is essential to highlight that the authors do not know whether any of the study participants had experienced neglect.

Each focus group began with a brief overview of the study, followed by an introductory game designed to establish a comfortable atmosphere for the young participants. Subsequently, basic guiding questions were introduced: “Do you think it is possible to recognize when a child suffers from neglect?” “And if so, how would you identify neglect?” “Who can help a child living in neglect?” The meeting concluded with a short feedback session to discuss the overall experience. 

### 3.3. Data Analysis

The discussions within the focus groups were recorded (the Arab sessions translated to Hebrew by professional speaker and the Arabic author ensured that no nuances lost in the translation), transcribed verbatim, and then anonymized. To analyze the data, we employed content analysis, describing what the participants expressed and adhering to the texts—using the participants’ own words. Following Charmaz’s [61] (2017) approach, we applied open coding to delineate key categories emerging from the data [59] (Denzin & Lincoln, 2011). The themes derived from the text were then organized based on the interconnections between the codes [62] (Corbin & Strauss, 2015). Finally, we compared the text from study participants across different groups, classifying responses according to the identified themes or domains—a standard analytical technique for comparing qualitative data [63] (Bernard et al., 2017).

To ensure the trustworthiness and rigor of the study, each member of the research team (Social Workers) independently read and re-read the texts multiple times to ensure a triangulated analysis [64] (Graneheim & Lundman, 2004). In instances of disagreement during the analysis, the authors engaged in discussions until a consensus was reached [65] (Guion et al., 2011).

### 3.4. Ethical Approval

The Ethics Committee of our academic institution granted approval for the study. Informed consent forms were signed by all participants and their parents. The focus group facilitator emphasized the youth’s right to withdraw from participation at any point during the session for any reason, following ethical guidelines [66] (Smith, 2011). While the study generally addressed the phenomenon without delving into specific personal situations, participants were offered referrals for professional assistance, given the issues’ severe sensitivity. Recordings and transcripts of group sessions were securely stored in a locked file on a password-protected hard drive. Furthermore, to preserve confidentiality, all participants’ names mentioned in quotations were substituted with pseudonyms [67] (Allen & Wiles, 2016).

## 4. Results

This chapter presents three themes that emerged from the analysis of the discussions in the 11 focus groups attended by young participants when they were asked about their ability to identify children suffering from neglect and about the factors that can help them. We found the findings very similar between the Arab and the Jewish participants.

The first theme refers to the signs that they believe allow them to identify children experiencing neglect, and the second theme to the challenges of identifying those children suffering from neglect. The third theme focused on which people or authorities may be addressed for assistance.

### 4.1. Theme 1: Signs of Identification for a Child Suffering from Neglect 

The study participants detailed symptoms that suggested a state of neglect, referring to the child’s feelings of isolation and alienation from society and social rejection. They referred to expressions of sadness and anger, inappropriate behavior and problematic behavior. They also referred to the child’s external appearance in terms of clothing and personal hygiene. An interesting topic that came up among some of the participants was the child’s self-neglect as a response to parental neglect.

#### 4.1.1. “He Has No Friends and Stays on the Streets Most of the Day and Walks around Alone”—Social Rejection and Alienation

The sub-theme that recurred most frequently about the emotional and social state of a neglected child was the sense of loneliness expressed by the child’s choice to be alone and not involved in the peer group but, at the same time, a feeling of being rejected by the peer group. The participants described expressions of sadness and anger as sometimes associated with the child’s apparent loneliness. For example, a Jewish boy describes: “The child feels neglected and sad. He is alone; he feels he has no one”. Meanwhile, an Arab boy said: “He’s someone who doesn’t like to get close to anyone and…when you look at him, you feel he has problems or he acts angry”. Thus, the participants of the different ethnic groups identify a neglected child as one whose behavior can be recognized by expressions of loneliness, sadness, and anger.

Some individuals have described introversion and a lack of involvement as signs of neglect within their peer group. One Jewish girl described: “A girl in our class is always alone and doesn’t like to participate in activities. I tried many times to talk to her, sometimes she would talk, and sometimes she wouldn’t”. A Jewish boy referred to the use of the cell phone as an expression of social isolation: “The boy is lonely in class, he is not in the mood, he only plays on the phone at school… he has no friends… he doesn’t communicate with others on the phone all day either… he is not used to making contact”. A girl from the same group added “… he is alone at home all the time”.

The participants pointed to the active rejection of a neglected child by the peer group, partly due to the “aura of neglect” received from him. An Arab boy shared: “We had a boy in class who had difficulties in his studies, and his grades were low, and the children laughed at him and teased him, and the child was always sad and his emotional state was difficult”. An Arab girl also spoke of the boy’s loneliness and described the disparaging attitude of the others: “How can I tell you…very lonely…very. They despised him… were strongly against him…” An Arab boy claimed that a neglected child suffers from both his friends and teachers: “He feels like a stranger among people; he feels that the students or the teachers do not accept him”.

#### 4.1.2. “… He’s Annoying… Does It on Purpose Because He Is Bored”—Inappropriate Behavior and Behavior Problems

The participants observed that as a response to the social rejection and alienation they experience, neglected children also display inappropriate and/or problematic behaviors. These behaviors sometimes stem from the need for attention, as one Jewish girl described: “I feel like I know of a neglected child at school; he always likes to talk and comment on trivial matters to attract attention because outside of the classroom or school, he doesn’t talk and no one is interested in him. He wants to make comments and be noticed”.

Other participants described reactions of fear by the neglected child, which are inappropriate to the situation. For example, the neglected child incorrectly interprets [being given a friendly slap], which is meant for fun: “You can see that the child is more scared. Let’s say you come to give the child a friendly pat or slap, and you see that he is so scared and wants to hide because he thinks you are coming to hit him”. An exaggerated fear response was described both concerning the peer group and to adults. For example, a religious Jewish boy explained: “I think that [neglected children] also feel insecure around adults. If they are yelled at or about to be punished, they react a little too much”. Jewish boys identified reactions of fear by neglected children in the presence of their parents, which are also accompanied by stress and silence: “I think that maybe a neglected child is reticent around his parents, he’s afraid, he doesn’t talk too much, he’s quiet and [‘lays low’]”.

In addition to this, the participants described signs of neglected children that manifest in dangerous behaviors. One of the participants in a group of Jewish boys described the need of the neglected child to fit in insistently with a group of boys even if it involves harmful and even dangerous behavior: “…a child who really tries to fit in forcefully, or a degenerate child, one who looks to follow others… a child at a young age will take cigarettes, drugs, alcohol”. In a group of boys from Arab society, they told of a neglected child who smokes to escape the reality in which he lives: “This neglected child seems to be running away from the neglect”. Both Jewish and Arab boys noted violent behavior among these children: “A child who hits other kids… maybe his older brothers beat him at home, and he learns from them to beat up those younger than him” and also: “[He] curses… yes, why don’t his parents teach him not to curse?”.

The children seem to see “behavioral problems” and/or “dangerous and violent behaviors” as signs that a child is suffering from neglect and interpret it as a result of a desire for social integration mixed with fear, lack of parental guidance and/or imitation of what is happening at home.

#### 4.1.3. “The Child Who Neglects Himself”—Self-Neglect as a Response to Parental Neglect

An interesting motif repeated in some of the participants’ descriptions in different focal groups was the description of “a child who neglects himself”, for example, by not listening to his parents or neglecting his studies. An Arab girl explained the behavior problems of the neglected child as part of family dynamics: “Because they neglect him, he pays them back, and he also neglects them by not listening to them… to get the message across”. Her classmate claims that when parents don’t care for the family’s livelihood: “… The child will have to go out to work, and then the child has responsibilities, but he needs to study and neglects himself”.

The participants in the groups described that the neglected child receives from the parents a kind of “message of neglect”, which he internalizes, as reflected in the quote of a Jewish boy who explained how this message could expand into a state of severe distress to the point of suicide:

“In such situations [of neglect], children commit suicide because they understand that if society has thrown them away and doesn’t want them, then at least they have a family. Moreover, if the family doesn’t want them, then who will want them? So, they give up on themselves just like society gave up on them, and even their parents did”.

This sub-theme reveals that the participants recognize a kind of “message/transmission” of neglect from the parents to the child and from the child to himself, his parents, and society. As the study participants described, this pattern exacerbates inner feelings of loneliness and despair.

#### 4.1.4. “The Way He Looks—Dirty, Torn Clothes, Torn Shoes”—Physical Neglect

Many of the study participants described signs of neglect manifested in clothing and the level of personal hygiene. The topic stood out in several statements in different focus groups. For example, religious Jewish girls said: “You see he is not the best groomed…” and also: “The way she dresses, I see the girl every day with a huge tear in her skirt…”. Arab boys added: “…from his appearance—if he is not clean and wears dirty and unfashionable clothes” or: “His smell is unpleasant”.

In addition to external appearance, the participants referred to suspicious signs they can identify in school equipment and its quality as evidence of neglect: “He has no books [or he has torn books] … he has no notebooks, no pencils…”. Others referred to identifying signs in the homes of neglected children, which include the parents’ negative attitude towards the child, lack and poverty: “In general, I think there is such a dark atmosphere, there are very few things to play with, and in general very few things that are related to the child. And let’s say the parent yells at his child to ask him to bring a cup of tea. Such a negative atmosphere” (Arab boy). A female participant in a group of religious Jewish girls added: “It’s really a mess; you can’t feel comfortable there. The refrigerator drawers are empty, as if no basic things exist. There are no toys. A torn couch, barely a bed to sleep on”. 

There was just a brief discussion in one of the focus groups, where participants expressed empathy for parents living in poverty who could not provide materials for their children, indicating that these cases were not considered neglect.

### 4.2. Theme 2: “You Don’t Know What’s Going on inside”—Challenges in Identifying Neglect

Quite a few statements by the participants of the different groups referred to the difficulty of identifying neglect situations. They referred to the disparity between what is visible “from the outside”, and what the boy/girl experiences from “inside”—inside the house and in the child’s inner world. For example, a religious Jewish boy explained: “There are houses that are very tidy, and you can’t see that they are neglected; you don’t know what’s going on inside; it could be that the parent hits the child and things like that”. Another participant in this group added: “It could be that the house is tidy and clean and everything is beautiful, it’s from the outside, and the inside you see that the parents don’t care much about the child”. The children seem to realize the possibility of an incongruence between the physical appearance of a house and the family dynamics that exist in it. Regarding aspects of the child’s appearance (such as sloppy and dirty clothing) that may identify neglect, we heard several voices who believed that it might not be neglect but a conscious choice of the boy not to groom himself. For example, a religious Jewish girl said: “I don’t think it’s about neglect, but maybe self-grooming”.

As with the difficulty of identifying child neglect due to possible disparity between the visibility of the home and the neglect of children taking place there, the group participants referred to the possible incongruence between the child’s inner feelings of neglect and his behavior in the peer group. An Arab girl claimed: “Not everyone who is neglected shows this… He smiles and looks like he doesn’t care, but we don’t know what’s inside him…”. Similarly, a religious Jewish boy stated: “…but he will try to hide it as much as possible so that they think he is a normal child; he does not want them to think of him as a neglected child”.

Along with the discrepancies between the outside appearance (the visible) and what is happening inside (the hidden), the young participants raised several reasons why the discrepancy may exist and the desire to hide the neglect. The concealment can stem from the child’s shame of knowing his situation and a desire to be considered normative but can also result from other reasons, such as the desire to protect his parents or his fear of them. A Jewish boy described it this way: “In a home where a child is beaten, he will not turn to anyone… and only he can decide whether to seek help… but he will not want to hurt them [the parents] because he loves them, but also because he is afraid of them”. This difficulty can be summed up in the expression of an Arab girl who said: “No one feels the pain of someone else”, and a Jewish participant who raised a concern that: “Maybe he’s just a boy that if I see him, I won’t really know…”.

This theme dealt with the difficulty of identifying signs of neglect from the peer group’s perspective. An interesting finding was the emphasis that the youths gave to the social–emotional aspect of a neglected child more than the physical aspect. In the social–emotional aspect, characteristics of feelings of loneliness, sadness, anger, rejection, and social alienation, maladjusted behaviors, and behavioral problems emerged. The participants interestingly referred to the child’s self-neglect, perhaps as a response to his parents’ neglect.

### 4.3. Theme 3: Appeal to Official and Unofficial Assistance

This theme refers to the official and unofficial agencies and people who can help a child suffering from neglect and to whom it is possible to turn from the point of view of the study participants. The participants presented a wide range of official and unofficial sources to which they could turn for assistance but also raised doubts about their ability to help the child suffering from neglect. They also addressed the possibility of a child suffering from neglect to help himself.

#### 4.3.1. “Yes, There Are Many… the School Counselor… the Teacher and Even the Classmates with Whom He/She Feels Comfortable”—People Who Can Be Sought Assistance

The participants indicated formal and informal sources to which they can turn for assistance and treatment when they identify a child suffering from neglect. They tend to view informal sources as more approachable than formal sources due to a lack of confidence in their ability to help and/or a fear of complicating a situation by involving formal sources.

As for the request for assistance from informal sources, many participants across all our focus groups raised the possibility for assistance within the nuclear and extended family: “the older brothers, the family, the uncles” and “if she has an older sister, her aunt, her grandmother”. Other suggestions were to turn to the peer group for help: “Whoever is around the child like friends”, “Call a good friend they have known for a long time”, as well as other figures in the child’s immediate environment: “A neighbor, a babysitter if you trust her. Someone close to the family. Not necessarily someone specific”.

Among the formal sources that may be addressed, the most referenced were educational figures (educator/teacher/counselor) in the child’s life: “The first thing for a teacher and a school counselor to think about how is to improve his situation” and in particular: “A teacher you trust, even if it is not your homeroom teacher” (religious Jewish girls). Group participants in the Arab sector also stated that it is possible to turn to a teacher, a school principal and/or a school counselor: “The teacher, the principal… anyone in the school can support him and help him…” In some groups, a clear priority was given to turning to a school counselor: “It’s better for a school counselor because you can ask her not to pass on the information you give her to anyone” (religious Jewish girl); “For example, I had a problem and I turned to a teacher and it helped…” (Jewish boy) “She can turn to a school counselor to ask for help. And the school counselor explains to her where to turn” (Arab boy).

It is important to note that only in four focus groups did the children refer to official bodies such as welfare services and the police or therapeutic frameworks in and outside the community. Some youths stated that they would have turned or referred to welfare, while others completely disagreed with such a position. Very few referenced mental health care workers as a potential resource for consultation, with one Jewish boy mentioning: “Perhaps a psychologist because he can know the reason for the neglect”. Jewish boys expressed apprehension toward the police and explained: “… because there are neighborhoods that have a ‘history’ with the police or welfare, who think that the police have a negative attitude toward them and everyone around them thinks that way, so it’s less correct to talk to them, it’s more correct to talk to those with whom they feel safe”. Others mentioned a lack of trust in the police and said: “It would be difficult for them to call the police”.

Other official sources suggested by participants in various focus groups were “youth centers” that children go to, educational supervisors, social workers, and child protective services, “working and learning youth clubs”, and “clubs and social workers”. Addressing after-school centers and clubs was raised as being similar to addressing a welfare office because there are social workers in the clubs. In these frameworks, the child can also receive assistance in purchasing clothing and find an opportunity to create friendships and a relaxing atmosphere.

In addition to settings in the community, there were references to possibilities in which the child leaves home. Thus, for example, a Jewish girl said:

If they are really neglected, then I actually think they should go somewhere else and leave the house. I know many places that combine the home with this framework; this way, it helps parents to be better parents…. It’s like there is another parent, someone who really cares for you. 

An Arab boy also commented on the possibility of being removed from home and said: “I suggest that they put him in an out-of-home service that will take care of him and give him all his rights. This framework will provide him with all his needs, such as food, clothing, education, etc. The framework can also provide him with emotional needs such as tenderness, warmth, and care”. 

One of the study participants noted: “Such a place may succeed in changing the child’s life for the better”.

In this sub-theme, the young participants identified a variety of sources in the child’s environment, formal and informal, to whom one can turn regarding parental neglect. They even brought up the possibility of removal from the home for the child’s benefit.

#### 4.3.2. “I Have Exactly Nothing to Do with It”—Hesitation about the Possibility of Assistance in a Situation of Neglect

The participants from different focus groups referred to the difficulty of helping with child neglect since the parents who are, in a certain sense, the ‘problem’ are also those who are responsible for the child. A Jewish boy emphasized: “In my opinion, it is not wise to contact the parents because parents who are neglectful do not know that they are neglectful”. Other participants mentioned the possibility that parents would become angry or threaten the child in case of external intervention. For example, Jewish boys said: “When you try to intervene, the child’s mother or father might threaten him”, or perhaps the child’s words will be perceived as unreliable when his parents are confronted: “…but after that, the parents will say that it’s just the child acting like that”. There is a reference here to the power gap between the child and his parents, expressed in fear of the parents as one of the signs of a child suffering from neglect, as mentioned in the previous theme.

Further, several young participants even referred to intervention in a situation of neglect as an alternative that may be problematic, perhaps damaging the delicate fabric of a family’s life. For instance, during a discussion among a group of Jewish boys, one of them said: “It depends on how serious it is; if it’s just something like… not that serious, then it’s not that important. You can talk to him, but you don’t need to turn to welfare services right now”. To which another participant replied: “If you are not sure, then it can create situations that are not exactly good”, and finally another participant emphasized:

“If I’m not sure what exactly is happening there, then it could be that according to what I tell them from my point of view, the welfare will do things, and in general inside the house there is a different reality, and I see one thing and inside they feel something else. So, I might cause this family to fall apart, even though in practice within the family, it was really fine”.

Several statements mentioned the lack of a recommended and suitable source to assist a neglected child. When considering reporting to an educator, an Arab girl expressed doubts about the educator’s ability to help, asking: “What can I say, what will the educator do? You can be in contact with welfare, you can talk to the parents… but what can she do?” A girl from a Jewish settlement also mentioned the difficulty of finding a suitable person who would be sufficiently involved in the child’s life and capable of dealing with the neglect:

“I actually think I could not be able to do anything about this kind of situation. If I see that he [the child] is experiencing neglect, but he’s not really related or close to me in some way, then there’s nothing I can really do. Even if I let my parents know, I don’t think they can do anything about this. It has to be someone from the outside, someone from within the family, someone random. A friend or acquaintance can’t do anything about it”.

Regarding the possibility of the study participants seeking assistance in cases where they have identified neglect, they sense a problem and mistrust in the ability of others to help, and risk of harming the delicate fabric of a family.

#### 4.3.3. The Child’s Self-Referral to Aid Agencies in Case of Neglected Child

The sub-theme that concludes this chapter discusses the issue of the ability of a neglected child to influence his situation, as suggested by the participants in the different focus groups. Few references were made to this possibility in the focus groups, but they were nevertheless interesting. One of the opinions that arose gave much space to the child’s recognition of his situation:

I think that if the child knows this by himself, if one of his friends tells him this, if I were in this situation, I will not go to some teacher or some other parent. What to do on his own because as soon as he is aware of this situation, he’ll find something to do; it really depends on him. If he feels it’s really neglect if others think it’s neglect but he doesn’t, well then…. but if he feels it’s neglect, then yes, he’ll find the right sources for himself. (Jewish girl).

Although this quote presents a position that believes in a child’s ability to influence his situation, it also reveals ambivalence, such as a possible disparity between the child’s perception and society’s perception of his situation.

Some participants alluded to the passive stance of a neglected child, attributing it to a lack of self-confidence: “Neglect is a force that erodes self-confidence”. Others suggested that the child’s acceptance of this situation stems from familiarity: “I believe the child may not feel compelled to seek assistance because it’s as if he was born into this reality. As far as he’s concerned, everything is fine, he doesn’t think there is a better reality, he doesn’t think there are better things—that’s what he knows”.

Following this quote that expresses a child’s lack of self-confidence and lack of awareness of his situation and/or the belief in the possibility of change, it should be noted that one of the groups in the Jewish sector had a discussion in which the boys viewed the child’s age as a factor that might influence the child’s acceptance or resistance to his situation: “The child is younger, and the parents can label him and say that this is how the child is, and he will, in turn, see himself that way, and his behavior will reflect their words”. The context of age came up among other participants who distinguished between the reaction of a young child and an older child who is more inclined to resist and rebel: “[He can] rebel against the parents, or he simply won’t ask them things… When he reaches the age of 15, then the father will no longer be able beat him because he will already be stronger than both the father and the mother”. From this, the participants show ambivalence about the child’s ability to change his situation, which is related to the child’s awareness, self-confidence, and age.

In this chapter, the participants shared that even if they could identify such children, they were ambivalent about the possibilities of their ability to influence the situation positively. They did mention, however, avenues of contacting official and unofficial figures in situations of neglect, as well as the possibility of removing the child to an out-of-home setting. Some of them even felt that under certain conditions, the child himself can bring about a change in his life.

## 5. Discussion

This study focused on the perceptions of youth regarding identifying signs of child neglect and turning to formal and informal aid sources in these situations. Through a thematic analysis of 11 diverse focus groups of Arab and Jewish youth, key themes emerged: characteristics of neglected children, the challenge in identifying child neglect, and the possibility vs. the improbability of turning to official and unofficial sources for assistance for a neglected child.

In the first theme, the young participants brought up a variety of signs according to which they recognize the neglect of children in their environment, and this is similar to the findings in the literature, which also refer to external signs (clothing and personal hygiene), lack of school equipment, games and equipment at home, behavior problems and social difficulties [1,27,28] (Daniel, 2008; Kobulsky et al., 2020; Maguire et al., 2015). The youths described signs of external neglect manifested in torn and dirty clothing and the level of personal hygiene, but also lack of school supplies and/or supplies in poor condition, lack of games, and neglect of the house in which the child lives (mess, a general lack of necessary goods, and a dilapidated facility).

In addition to these signs, the young participants offered clues to recognizing child neglect that has not been mentioned in the literature of neglect, highlighting the contribution of the research and the importance of paying attention to children’s perceptions. They identified children who suffer from neglect as those who choose to distance themselves from their peers and isolate themselves and who are often mutually rejected by their peers. They also noted that children who tend to neglect themselves express the neglect they experience from their parents and friends. In general, the youths emphasized the emotional (anger, sadness, loneliness), behavioral (behavior problems, self-neglect), and social (rejection, avoidance, and social alienation) signs as helping them identify children suffering from neglect. At the same time, the professional literature refers only in a limited way, if at all, to these signs.

In the second theme, the young participants referred to the difficulties and challenges in identifying situations of child neglect, similar to the general agreement in the professional literature that emphasizes the difficulty and elusiveness of the phenomenon of neglect. Disagreement regarding the elements of the definition of child neglect and what should be included in them makes it difficult for professionals and the general public to identify these cases [24] (Hamarman et al., 2002). It is difficult to define when a child’s care is adequate and when it is not, so professionals often allege child neglect only in the most serious cases when the child’s safety is at risk but gloss over cases in which the child’s emotional well-being is at risk [25] (DeLong-Hamilton et al., 2016;). Another identification challenge is the dependence on subjective perspectives and the differences among professionals in naming neglect [11] (Elias et al., 2018).

The participants explained the difficulties in identifying the phenomenon by saying that sometimes what appears to be OK “from the outside” (a well-kept home or a child who tries to show that he is fine outwardly) does not necessarily sync with what is happening “inside” (family dynamics of neglect or a child who suffers from neglect). Therefore, they can only sometimes trust what they see to detect child neglect. Professionals also find it challenging to define what they call “reasonable suspicion” of child neglect among the families in their care [42] (Levi et al., 2015). 

The youths also offered reasons why children who suffer from neglect hide their situation, not only out of shame and unwillingness to be pitied but also fear of the parents and reluctance to harm them and the integrity of the family. The need to protect the parents from authorities and possible punitive measures, as well as the fear of the consequences to their own lives and the integrity of their family, was also discussed in a study that investigated the conduct of children suffering from neglect [49] (Lavi & Katz, 2016).

The third theme dealt with the youths’ views on reporting to community authorities when they identify situations of children experiencing neglect. Building on the previous theme in which they feared cooperation with official bodies entrusted with protecting the child, the youth preferred turning to informal bodies within the nuclear and extended family. Previous findings also indicated that children prefer to seek help from friends in cases of abuse and neglect [68] (Vincent & Daniel, 2004), as shown in the research literature in the context of the need for cultural sensitivity regarding the reporting and disclosure of child neglect at home. For example, a literature review examining global challenges in child neglect found that in Asian countries, one of the barriers to preventing child neglect is the emphasis on family privacy [1] (Kobulsky et al., 2020). In Palestinian society, there is resistance to the involvement of officials in the family framework and an assumption that external factors do not better understand the well-being of the family’s children and their education than the parents [69] (Haj-Yahia, 1995). Similarly, in Saudi Arabia, a study found that reporting child neglect to officials is still not accepted in society [18] (Alyoubi et al., 2024).

The participants specifically mentioned people in the educational frameworks, such as the principal/educator/school counselor/teacher, as the appropriate “authorities” for reporting child neglect, but at the same time, expressed doubt that they could effectively offer a solution to the child’s situation. Similarly, the research literature supports that those in the education system who have direct and daily contact with the children are in a good and vital position to identify cases of child neglect and report them [27,70] (Daniel, 2008; Sharley, 2022), even if in practice obstacles may prevent them from reporting to child protection services [71,72] (Alazri & Hanna, 2020; Goebbels et al., 2008). Few of the participants mentioned seeking assistance from welfare officials and police, and most expressed fear of reporting to those officials in the community. This fear of reporting to the police has been previously documented in the professional literature in Israel as a characteristic of minority communities that prefer to solve such issues within the community with the help of leadership rather than involving the police [73] (Roer-Strier & Nadan, 2020). Some youths suggested contacting care providers in out-of-home settings, as in these cases, they saw the possibility of removing the child from the home for his benefit.

The youth attitude to the possibilities of helping children suffering from neglect was mainly expressed in their hesitation. Addressing the child’s parents was seen as problematic for fear that more significant harm would be caused to the child if the parents realized that their behavior had been exposed. A similar concern was raised in a study conducted in Saudi Arabia when 63.4% of the participants expressed concern that reporting the situation could have adverse repercussions or lead to violence against the child [18] (Alyoubi et al., 2024). Another question arose regarding how professionals perceive the severity of neglect compared to how youths perceive it. This disparity could lead to professionals not taking the situation seriously or acting on it. Studies do show the disparity between professionals and the public in terms of the perceptions of the severity of a problem [74] (Marey-Sarwan & Meir, 2020). Furthermore, as already noted, there is a great deal of subjectivity among professionals regarding the definition of child neglect.

Finally, it should be noted that a minority of youths in this study raised the possibility that the child suffering from neglect could turn to aid sources on his/her own. At the same time, they raised doubts over the possibility of self-reporting because of different perceptions between the child and those around him/her (i.e., he/she does not feel that his situation requires assistance), because of his dependence upon his parents, and from the effect of the child’s age (mature children may report more about their situation). This raises the question of how the welfare system can make sure that a child feels safe to report neglect. For example, it is possible to see increasing use of text messages to contact phone lines for reporting, in which children are a small minority of the callers [75] (Ortiz et al., 2021). Projects do exist that allow children to report being distressed and/or harmed via easily accessible means where professionals and volunteers are on standby to respond to them quickly (for example, hotlines).

## 6. Summary and Implications

While the findings of this study partially confirm the information available in the literature dealing with child neglect, they also reveal how other youths detect signs for identifying children experiencing neglect by emphasizing emotional, behavioral, and social signs more than external appearance (clothing and hygiene) and parental behavior (i.e., supervision). 

This study also contributes to the practice of those dealing with the child neglect phenomenon in several ways: First, the need to create an opportunity for youth to take part in the definition of child neglect to clarify the attitudes of the public and welfare services toward the phenomenon. Second, to encourage those in the educational frameworks perceived by youth as a central address for reporting to deal with challenges and obstacles in addressing child protection authorities. It seems that additional training and/or a change in the reporting policy of education officials is required to make them an even more significant address for children, especially since they are the ‘external’ figures most present in a child’s environment. Third, there is a need to establish acceptable standards for neglect among youths and professionals. Youhts must turn to child protection agencies and trust that they will act to resolve the situation and that their process will be continuous, professional, and optimal. Fourth, consideration must be given to the cultural competence required to encourage reporting among populations in whose culture it is not acceptable to involve formal parties in issues such as child neglect. Also necessary is to assure the general public that child protection agencies are doing as much as possible to prevent harm to children and keep their best interests in mind during the intervention. We believe that implementing these practical recommendations will contribute to increasing the number of reports and referrals for assistance in situations of child neglect among the youth, the public as a whole, and professionals who are not part of the child protection system. An increase in reports and requests for assistance can save children from serious, long-term harm that can adversely affect their continued development.

## 7. Limitations

This study also has some limitations. First, the study focuses on a relatively small population of youths from the northern periphery of our country. Although an effort was made to include children from different religions and different religious levels, the numbers do not necessarily reflect Israeli society. The study focused on a specific age range of youths, and it is possible that younger children would respond differently to our questions and discussions. Therefore, future studies may involve participants of other age groups. At the same time, our study examined the points of view of young people who are often less researched, though a youth participatory action research might be a good fit, allowing the youth to be involved in shaping the research and the recommendations in a deeper way.

## Data Availability

The original contributions presented in the study are included in the article, further inquiries can be directed to the corresponding author/s.
